# Pelvic floor muscle training in managing female stress urinary incontinence

**DOI:** 10.6026/973206300221632

**Published:** 2026-03-31

**Authors:** Sanjay P Dhangar, Nisha B Jain

**Affiliations:** 1 Department of Urology, Bharati Hospital & Research Centre, BVDTU, Pune, Maharashtra, India; 2 Department of Surgery, SKS Hospital Medical College and Research Centre, Mathura, Uttar Pradesh, India

**Keywords:** Stress urinary incontinence (SUI), pelvic floor muscle training (PFMT), women's health, pelvic floor strength, quality of life

## Abstract

Stress urinary incontinence (SUI) in women remains a prevalent condition, leading to significant physical and emotional distress, often lacking effective non-invasive treatment options. Therefore, it is of interest to evaluate the effectiveness of pelvic floor muscle training (PFMT) for managing stress urinary incontinence (SUI) in women. Hence, a total of 100 women underwent a 12-week PFMT program, with assessments of urinary leakage, pelvic floor muscle strength and quality of life. Significant improvements were noted in all outcome measures, including reduced leakage, enhanced muscle strength and better quality of life. The study enhances current understanding by demonstrating significant improvements in urinary leakage, pelvic floor muscle strength and quality of life, contributing valuable insights into non-surgical management of SUI.

## Background:

Stress urinary incontinence (SUI) is a common and distressing condition among women, characterized by the involuntary leakage of urine during activities that increase intra-abdominal pressure such as coughing, sneezing, laughing, lifting, or physical exertion. It represents a significant public health concern due to its high prevalence, chronic nature and substantial impact on physical, psychological and social well-being [1]. Although not life-threatening, stress urinary incontinence can severely compromise a woman's quality of life, leading to embarrassment, social withdrawal, reduced participation in physical activity and negative effects on sexual health and self-esteem [2]. The etiology of stress urinary incontinence is multifactorial, with pelvic floor muscle weakness being one of the primary underlying mechanisms. The pelvic floor muscles play a crucial role in supporting pelvic organs and maintaining urethral closure pressure during increases in intra-abdominal pressure. Damage or weakening of these muscles often resulting from pregnancy, vaginal childbirth, aging, obesity, menopause, chronic coughing, or heavy physical labor can impair their ability to provide adequate support to the bladder neck and urethra, leading to urine leakage [3]. As women age and hormonal changes occur, particularly the decline in estrogen levels, the connective tissues and muscles of the pelvic floor may further lose strength and elasticity, exacerbating symptoms of stress urinary incontinence [4]. The prevalence of stress urinary incontinence varies widely across populations, with studies reporting that up to one in three adult women experience some degree of urinary incontinence during their lifetime. Despite its high prevalence, many women do not seek medical help due to stigma, lack of awareness about treatment options, or the misconception that urinary leakage is a normal and inevitable consequence of aging or childbirth [5]. This underreporting contributes to delayed diagnosis and management, allowing symptoms to persist or worsen over time. Consequently, there is a growing emphasis on early identification and conservative management strategies that are safe, cost-effective and acceptable to women [6]. Management options for stress urinary incontinence include conservative, pharmacological and surgical interventions. Among these, conservative management is widely recommended as the first-line approach, particularly for mild to moderate cases [7]. Pelvic floor muscle training (PFMT) has emerged as the cornerstone of conservative treatment for stress urinary incontinence. PFMT involves the systematic and repetitive contraction and relaxation of the pelvic floor muscles with the aim of improving muscle strength, endurance, coordination and reflex activity. By enhancing pelvic floor support and urethral closure mechanisms, PFMT can reduce or eliminate urine leakage during activities that increase intra-abdominal pressure [8].

Pelvic floor muscle training offers several advantages that make it an attractive therapeutic option. It is non-invasive, low-cost and associated with minimal adverse effects. Additionally, PFMT empowers women by actively involving them in their own care and promoting long-term self-management [9]. Numerous clinical studies have demonstrated that regular and correctly performed PFMT can significantly reduce the frequency and severity of stress urinary incontinence episodes and improve quality of life. However, the effectiveness of PFMT largely depends on proper technique, adherence to the exercise regimen and the duration and intensity of training [10]. Despite strong evidence supporting PFMT, challenges remain in its implementation and evaluation. Many women have difficulty correctly identifying and contracting their pelvic floor muscles, which can limit the effectiveness of training. Lack of supervision, inadequate instruction, poor motivation and inconsistent follow-up are common barriers to successful outcomes [11]. Furthermore, variations in training protocols, including differences in exercise frequency, duration, supervision methods and outcome measures, make it difficult to compare results across studies and to establish standardized guidelines applicable to diverse populations [12]. Assessment of pelvic floor muscle training outcomes is therefore essential to determine its true effectiveness in the management of stress urinary incontinence. Objective measures such as pelvic floor muscle strength, endurance and functional performance, along with subjective measures including symptom severity and quality of life, provide valuable insights into treatment efficacy [13]. Evaluating these parameters before and after intervention helps clinicians and researchers understand the extent of improvement attributable to PFMT and identify factors influencing treatment success. Such assessments are particularly important in resource-limited settings, where conservative therapies like PFMT may represent the most feasible and accessible treatment option [14]. In addition, cultural, socioeconomic and educational factors can influence awareness, acceptance and adherence to pelvic floor muscle training programs. Assessing PFMT in different populations allows for the development of tailored interventions that address specific needs and barriers faced by women. This approach can enhance patient engagement, improve compliance and ultimately lead to better clinical outcomes [15]. Therefore, it is of interest to report determine the effectiveness of pelvic floor muscle training in improving pelvic floor muscle function and reducing symptoms of stress urinary incontinence among women.

## Methodology:

## Study design:

This original research was designed as a prospective interventional study to assess the effectiveness of pelvic floor muscle training (PFMT) in the management of stress urinary incontinence among women.

## Study setting:

The study was conducted in the Department of Obstetrics and Gynecology / Physiotherapy (Pelvic Health Unit) of a tertiary care hospital over a period of 6 months.

## Study population and sample size:

A total of 100 women diagnosed with stress urinary incontinence were recruited for the study. The sample size was determined based on feasibility, outpatient attendance and previous similar studies reporting significant improvement with pelvic floor muscle training.

## Inclusion criteria:

[1] Women aged 20-60 years

[2] Clinically diagnosed cases of stress urinary incontinence

[3] Symptoms present for at least 3 months

[4] Ability to understand and perform pelvic floor muscle exercises

[5] Willingness to participate and provide written informed consent

## Exclusion criteria:

[1] Mixed or urge urinary incontinence

[2] History of pelvic surgery within the last 6 months

[3] Neurological disorders affecting bladder function

[4] Active urinary tract infection

[5] Pregnancy

[6] Severe pelvic organ prolapse (Grade III or IV)

## Sampling technique:

Participants were selected using a purposive sampling technique from women attending the outpatient department who fulfilled the inclusion criteria.

## Baseline assessment:

At baseline, all participants underwent a detailed assessment, including:

[1] Demographic data (age, parity, body mass index, occupation)

[2] Obstetric and gynecological history

[3] Duration and severity of stress urinary incontinence

## The severity of urinary incontinence was assessed using:

[1] A validated urinary incontinence questionnaire (*e.g.*, International Consultation on Incontinence Questionnaire- Short Form)

[2] Frequency of urine leakage episodes per week

[3] Pad usage per day

Pelvic floor muscle strength was assessed using the Modified Oxford Grading Scale through digital vaginal palpation.

## Intervention:

## Pelvic floor muscle training program:

All participants were enrolled in a structured pelvic floor muscle training program for 12 weeks. Prior to starting the exercises, participants received individual instruction on correct identification and contraction of pelvic floor muscles. Education was provided using verbal explanation, diagrams and demonstration.

## The PFMT protocol included:

[1] Slow contractions: holding pelvic floor muscle contraction for 5-10 seconds followed by equal relaxation

[2] Fast contractions: quick maximal contractions followed by immediate relaxation

[3] Each session consisted of 10 slow contractions and 10 fast contractions

[4] Exercises were performed three times daily

Participants were advised to perform the exercises in different positions (lying, sitting and standing) as training progressed. Compliance was encouraged through exercise diaries and periodic reinforcement during follow-up visits.

## Follow-up and monitoring:

Participants were followed up at 4-week intervals (4, 8 and 12 weeks). At each visit, proper exercise technique was reinforced, adherence was reviewed and difficulties were addressed.

## Outcome measures:

Primary outcome measures included:

[1] Reduction in frequency of urinary leakage episodes

[2] Improvement in pelvic floor muscle strength (Modified Oxford Scale)

## Secondary outcome measures included:

[1] Improvement in quality of life scores

[2] Reduction in pad usage

All outcome measures were reassessed at the end of 12 weeks and compared with baseline values.

## Ethical considerations:

Ethical approval was obtained from the Institutional Ethics Committee prior to the commencement of the study. Written informed consent was obtained from all participants. Confidentiality and anonymity of participant data were maintained throughout the study.

## Statistical analysis:

Data were entered into Microsoft Excel and analyzed using statistical software. Continuous variables were expressed as mean ± standard deviation, while categorical variables were expressed as frequencies and percentages. Pre- and post-intervention comparisons were made using paired t-tests or Wilcoxon signed-rank tests, as appropriate. A p-value of <0.05 was considered statistically significant.

## Results:

A total of 100 women with stress urinary incontinence completed the 12-week pelvic floor muscle training (PFMT) program and were included in the final analysis. All participants demonstrated good compliance with the prescribed exercise regimen. At baseline, the majority of participants were multiparous women with a mean duration of stress urinary incontinence of more than one year, indicating a chronic condition prior to intervention (Table 1 - see PDF). A marked reduction in the frequency of urinary leakage episodes was observed following 12 weeks of PFMT. The mean weekly leakage episodes reduced by approximately 67%, indicating substantial clinical improvement (Table 2 - see PDF). At baseline, most participants demonstrated weak pelvic floor muscle strength (Grades 1-2). Post-intervention assessment showed a clear shift toward higher strength grades, with 80% of women achieving Grade 3 or above after PFMT (Table 3 - see PDF). Significant improvement was noted in quality of life scores, with a reduction in mean ICIQ-SF score by more than 50%. Pad usage also decreased substantially after the intervention period (Table 4 - see PDF). STATA analysis demonstrated statistically significant improvements in all primary and secondary outcome measures following pelvic floor muscle training. The reduction in urinary leakage episodes, improvement in pelvic floor muscle strength and enhancement in quality of life scores were all highly significant (p < 0.001) (Table 5 - see PDF). Overall, the results indicate that pelvic floor muscle training led to significant clinical and functional improvements among women with stress urinary incontinence. The intervention was effective in reducing urinary leakage frequency, strengthening pelvic floor muscles, decreasing pad usage and improving quality of life, as supported by both clinical assessment and STATA statistical findings.

## Discussion:

The findings of this study demonstrate that a 12-week structured pelvic floor muscle training (PFMT) program significantly improved pelvic floor muscle strength, reduced the frequency of urinary leakage, diminished pad usage and enhanced quality of life among women with stress urinary incontinence (SUI). These results are consistent with and contribute to the growing body of evidence supporting PFMT as an effective first-line conservative treatment for SUI. In the present research, participants showed marked improvements in objective and subjective measures after PFMT, reflected by significant gains in pelvic floor muscle strength grades and reductions in urinary leakage episodes. These outcomes align with the results of Kucukkaya and Sut (2021), [16] where pelvic floor muscle training with or without additional abdominal exercises produced significant improvements in pelvic floor activity and symptom measures among women with SUI. In that randomized controlled trial involving 64 women, PFMT groups experienced significant reductions in symptoms and distress related to urinary incontinence, underscoring the effectiveness of targeted exercise interventions in managing stress urinary leakage. Similarly, Fitz *et al.* (2020) [17] compared PFMT administered in outpatient versus home settings among women with predominant SUI. Although outpatient PFMT yielded higher cure rates than home exercises, both intervention arms experienced reductions in urinary leakage and improvements in pelvic floor muscle function over three months. This corroborates the notion that regular, structured PFMT whether supervised or guided at home can offer meaningful therapeutic benefits, reaffirming the improvements observed in this study's cohort. The role of adjunctive methods in PFMT has also been assessed in other research. Wang *et al.* (2024) [18] conducted a large multicenter randomized trial comparing PFMT alone versus PFMT supplemented with a home-based pressure-mediated biofeedback device in women with postpartum SUI. Both groups improved, but the biofeedback-assisted group showed greater reductions in incontinence severity and higher cure rates, suggesting that additional feedback mechanisms may enhance PFMT outcomes. While this study did not include biofeedback, our positive results without adjunctive devices highlight that PFMT alone remains a powerful intervention, yet adaptations like biofeedback might optimize outcomes further. Older RCTs reinforce the present findings as well. In Hirakawa *et al.* (2013) [19], PFMT performed with or without biofeedback effectively reduced symptoms of SUI and improved quality-of-life domains, with no significant added benefit from biofeedback at short-term follow-up. This study supports the basic premise that structured; repeated PFMT is effective for SUI management, consistent with the significant strength and symptom improvements seen in the current research. Additionally, the broader evidence synthesis reported by Alouini *et al.* (2022) [20] in a systematic review of PFMT with or without adjunctive therapies reveals that PFMT significantly reduces urinary incontinence and enhances pelvic floor muscle contraction in non-pregnant women. Their review of multiple RCTs confirms that PFMT whether alone or combined with methods like biofeedback or electrostimulation produces clinically meaningful improvements compared to control groups. These findings support the consistent efficacy of PFMT demonstrated in our results and highlight the robustness of PFMT across diverse clinical settings and populations. More recent evidence further strengthens this interpretation. Bharti *et al.* (2024) [21], in a systematic review of randomized controlled trials, concluded that pelvic floor muscle training is effective in reducing symptoms of stress urinary incontinence and improving quality of life, whether delivered alone or in combination with adjunctive approaches. Importantly, their review suggests that a minimum of 12 supervised PFMT sessions performed three times per week may constitute an effective first-line regimen for women with SUI. This is highly relevant to the present study, as the significant post-intervention improvements observed after 12 weeks of structured PFMT support the practicality and effectiveness of a similarly sustained training approach.

In addition, Dimli *et al.* (2024) [22] compared PFMT with modified Pilates exercises in elderly women with stress urinary incontinence in a randomized clinical trial. Both groups demonstrated significant improvements in incontinence severity, symptom distress and quality-of-life measures after 12 weeks, while PFMT showed greater improvement in electromyographic indicators of pelvic floor muscle activation. These findings are consistent with the present results and further indicate that exercise-based conservative interventions are beneficial across different age groups and clinical contexts. At the same time, the superior pelvic floor activation seen with PFMT in that study supports the view that targeted pelvic floor training may offer more specific neuromuscular benefits than broader exercise-based approaches. Compared to these previous studies, this research adds value by reinforcing that even without advanced adjunctive tools, a well-supervised and structured PFMT program yields statistically and clinically significant improvements in muscle strength, symptom burden and quality of life. The magnitude of improvement in the present study rivals or exceeds that reported in several trials, suggesting that adherence and proper instruction ensured through follow-up and reinforcement may be key determinants of PFMT success. Moreover, the use of validated measures, comparative pre-training and post-training assessments and statistically robust analyses strengthen confidence in these findings. In summary, the present study's outcomes are congruent with previous PubMed-indexed research, reinforcing the evidence that pelvic floor muscle training is an effective, non-invasive strategy for managing stress urinary incontinence in women. PFMT consistently improves pelvic floor muscle function and reduces incontinence symptoms, supporting its role as essential conservative therapy. Future research might explore the long-term sustainability of these benefits and strategies to improve adherence and accessibility across diverse populations.

## Conclusion:

Pelvic floor muscle training is an effective and safe conservative intervention for women with stress urinary incontinence. A structured 12-week program improves pelvic floor muscle strength, reduces urinary leakage and pad use, and enhances quality of life. Thus, we show pelvic floor muscle training as a practical, non-invasive, and cost-effective first-line treatment for mild to moderate stress urinary incontinence.
